# Use of a perforator-based flap from the ulnar palmar artery for the repair of defects caused by the treatment of severe contractures due to Dupuytren's disease

**DOI:** 10.3389/fsurg.2024.1508100

**Published:** 2025-01-23

**Authors:** Jiangping Dai, Hui Wang, Haifeng Wang, Xinyang Sun, Yihan Zhang

**Affiliations:** ^1^ Department of Hand Surgery, Tangshan Second Hospital, Tangshan, China; ^2^ College of Graduate School, North China University of Science and Technology, Tangshan, China

**Keywords:** Dupuytren's contracture, ulnar palmar artery, surgical flap, perforator flap-based, wound repair

## Abstract

**Objective:**

The aim of this study was to investigate the clinical effectiveness of a perforator flap from the ulnar palm in repairing soft tissue defects in the palm and fifth digit following severe Dupuytren's contracture.

**Methods:**

A retrospective study was conducted between March 2013 and January 2023. In total, 25 patients with soft tissue defects that occurred after severe Dupuytren's contracture release were treated using a perforator flap from the ulnar palm. Improvements in symptoms and functionality were assessed and compared before and after surgery. Data regarding the degree of contracture, two-point discrimination, and the total active motion (TAM) scale of the affected hand were collected by the same physician at the specified time points (preoperative, postoperative, and final follow-up), along with an evaluation of the donor and recipient areas of the flap. Concurrently, patients completed the visual analog scale (VAS) and disability of arm, shoulder, and hand (DASH) questionnaires.

**Results:**

All 25 flaps survived uneventfully, and the wound healed primarily. The follow-up time was 15–26 months (average, 20.48 months). At the final follow-up, the patient satisfaction score related to flap appearance ranged from 4 to 5 (mean, 4.72). The average two-point discrimination value of the flap was 5.52 ± 0.97 mm. The contracture of the affected hand was improved with a mean TAM of 229° ± 16.75°. Furthermore, the degree of pain and functional status of the affected hand had improved (*P* < 0.05).

**Conclusion:**

A perforator flap from the ulnar palm is a good option to repair the wound that occurs after severe Dupuytren's contracture release.

## 1 Introduction

Dupuytren's contracture is a common localized fibrotic condition that often affects the fourth digit and fifth digit. It is more prevalent in Northern Europe, typically starting between the ages of 50 and 70, with men being three times more susceptible than women (1). Initially, firm nodules develop on the palms of the hand, which later evolve into fibrous collagen cords extending toward the fingers (2, 3). As the condition progresses, these cords thicken, mature, and contract, leading to permanent finger flexion deformity. The patient's table top test is positive. Surgical intervention is often necessary for individuals who sustain severe Dupuytren's contracture. The severity of Dupuytren's contracture is graded based on the Tubiana staging system, with severe cases falling under Tubiana stages Ⅲ and IV (4). In cases of severe contracture, local soft tissue defects with exposed tendons, nerves, and vessels may occur after removing the diseased palmar fascial tissue. These defects should be promptly repaired with a suitable flap (5).

The perforators of the ulnar palmar artery in the fifth digit are situated approximately 1.3 cm above the head of the fifth metacarpal bone. They travel between the superficial hypothenar tendon and the fifth metacarpal bone subcutaneously, forming connections directly or indirectly with the descending dorsal branch of the ulnar artery. Along its path, small blood vessels branch out to supply nearby soft tissues (5). A perforator flap from the ulnar palmar digital artery of the fifth digit is a suitable option for repairing local soft tissue defects on the palm and the fifth digit (6).

This study aims to investigate the design of a perforator flap based on the ulnar palmar artery perforator of the fifth digit for repairing soft tissue defects in the palm and fifth digit.

## 2 Materials and methods

In this study, patients who met the following criteria were included: (1) patients suffering from Dupuytren’s contracture in Tubiana stage III or IV; (2) patients with a soft tissue defect of the palm after surgery and repaired using a perforator flap from the ulnar palmar artery. (3) Age and gender were not limited. The exclusion criteria were as follows: (1) clinical data were incomplete; (2) patients with severe scar constitution and diabetes; (3) patients with Raynaud syndrome.

All the procedures in this study were approved by the Ethics Committee of the Second Hospital of Tangshan and in accordance with the ethical standards of the Institutional Research Committee and with the Declaration of Helsinki. All patients signed a written informed consent form for the operation.

### 2.1 Surgical technique

**Figure 1:**
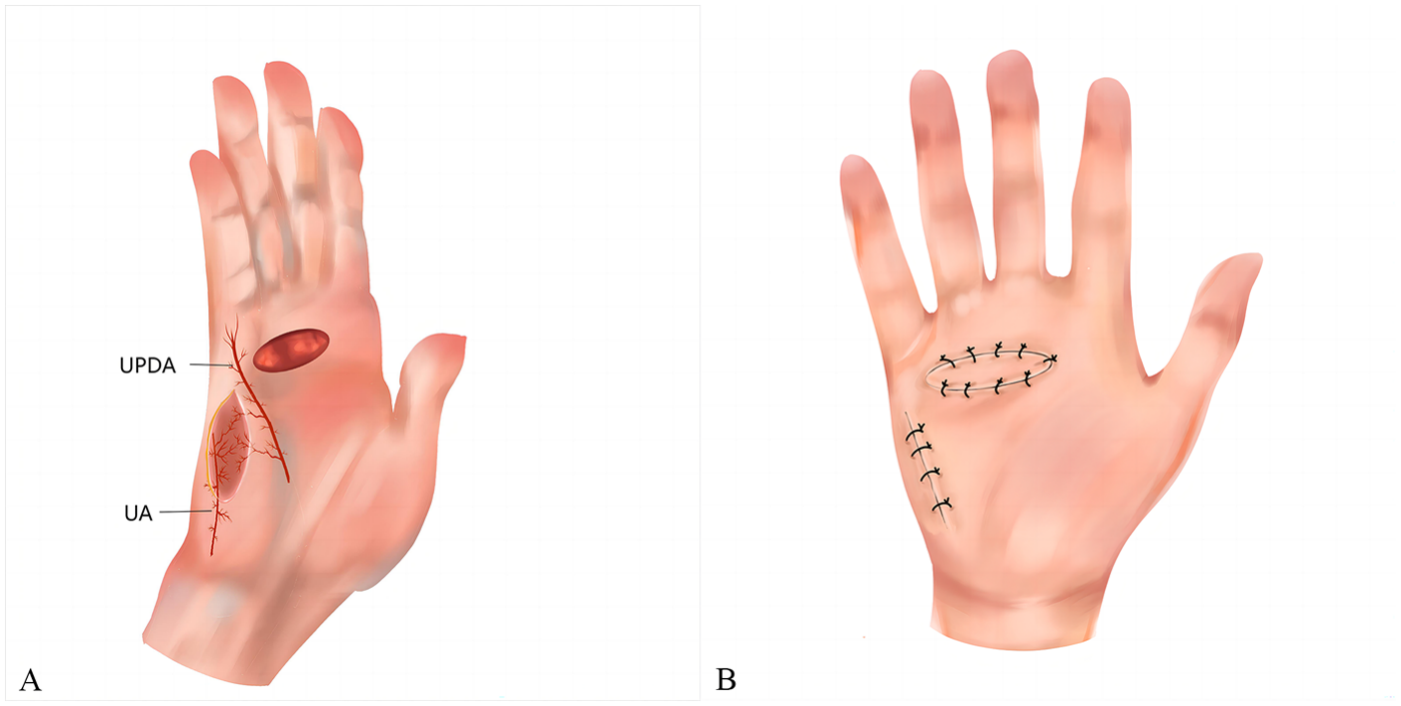
**(A)** The perforator flap was harvested at the ulnar palm and pedicled through the perforator of the ulnar palmar digital artery of the little finger. **(B)** The wound was repaired with the perforator flap and the donor site was closed directly. UPDA, ulnar palmar digital artery; UA, ulnar artery.

The ulnar palmar artery was preoperatively identified by high-frequency Doppler ultrasound and marked on the surface of the ulnar palm. The patient was placed in the supine position and underwent surgery under a brachial plexus nerve block with tourniquet control. Loupe magnification and an operating microscope were also needed. Following hand disinfection, the contracture site was palpated and the incision location was labeled. Subsequently, the skin, subcutaneous tissue, and fascia were incised along the scar marks on the palm. Care was taken not to injure the deep nerves and arteries when cutting the contracture band. Even after the complete removal of the contracture tissue, flexion of the affected finger may still be observed. To address this, an incision near the proximal interphalangeal (PIP) joints was designed to release the joints. The resected contracture tissue was pathologically examined. Skin and soft tissue defects, with exposed nerves, tendons, and vessels, were visible after the dissection. These defects, located either on the ulnar side of the palm or the palmar side of the fifth digit, were repaired using a perforator flap of the ulnar palm. The perforator flap of the ulnar palmar digital artery was designed according to the size, shape, and position of the wound. The rotation point of the flap is usually located around the metacarpophalangeal (MP) joint. The axis of the flap generally aligns with the line between the pea bone and the midpoint of the hypothenar striae. The flap size should be greater than 10%–20% of the wound dimensions. The ulnar margin of the flap was first incised, identifying the perforator entering the flap from the ulnar palmar digital artery of the fifth digit (Figure 1A). During the cutting of the flap pedicle, 0.5 cm of fascial tissue around the perforator was preserved on both sides of the axis. The flap was dissected from the superficial layer of the hypothenar muscle from the proximal to the distal up to the rotation point. The tourniquet was then released, ensuring normal blood supply to the flap. The flap was transferred to cover the wound through the open tunnel which was formed between the donor and recipient sites. The flap donor site was directly sutured (Figure 1B).

Postoperative management included drainage strip placement, sterile dressing application, and elevation of the affected limb to prevent venous congestion. Skin flap color, temperature, and capillary refill were monitored closely for 7 days. Stitches were removed after 2 weeks, followed by the rehabilitation exercises with the aid of a physical therapist.

### 2.2 Evaluation of outcomes

Data acquisition and assessment were performed by a single senior hand surgeon (HuW). All patients completed the visual analog scale (VAS) and disability of the arm, shoulder, and hand (DASH) questionnaires preoperatively, postoperatively, and at the final follow-up (7). Concurrently, the MP and PIP joint contracture levels and the two-point discrimination of the flap were assessed by the same physician. Furthermore, the appearance of the flap and donor site was evaluated based on the Michigan Hand Outcomes Questionnaire (MHQ) (very satisfied = 5 points, satisfied = 4 points, average = 3 points, dissatisfied = 2 points, very dissatisfied = 1 point) (8). At the final follow-up, the total joint motion of the patient post-treatment was assessed using the total active motion (TAM) scale. The TAM scores of the affected hands are classified as excellent for more than 220°, as good for 180°–220°, and as poor for less than 180°.

Data were analyzed with the SPSS 25.0 statistical software (IBM, Armonk, NY, USA). A paired *T*-test was conducted to compare the recovery of the affected fingers at the final follow-up with the degree of contracture before surgery. It was also conducted to compare the contracture degree of the affected fingers at the final follow-up with the healthy contralateral hand. The Kruskal–Wallis (K–W) test was used to compare the VAS and DASH scores at the final follow-up, after surgery, and before surgery. *P* < 0.05 was considered statistically significant.

## 3 Typical case

**Figure 2:**
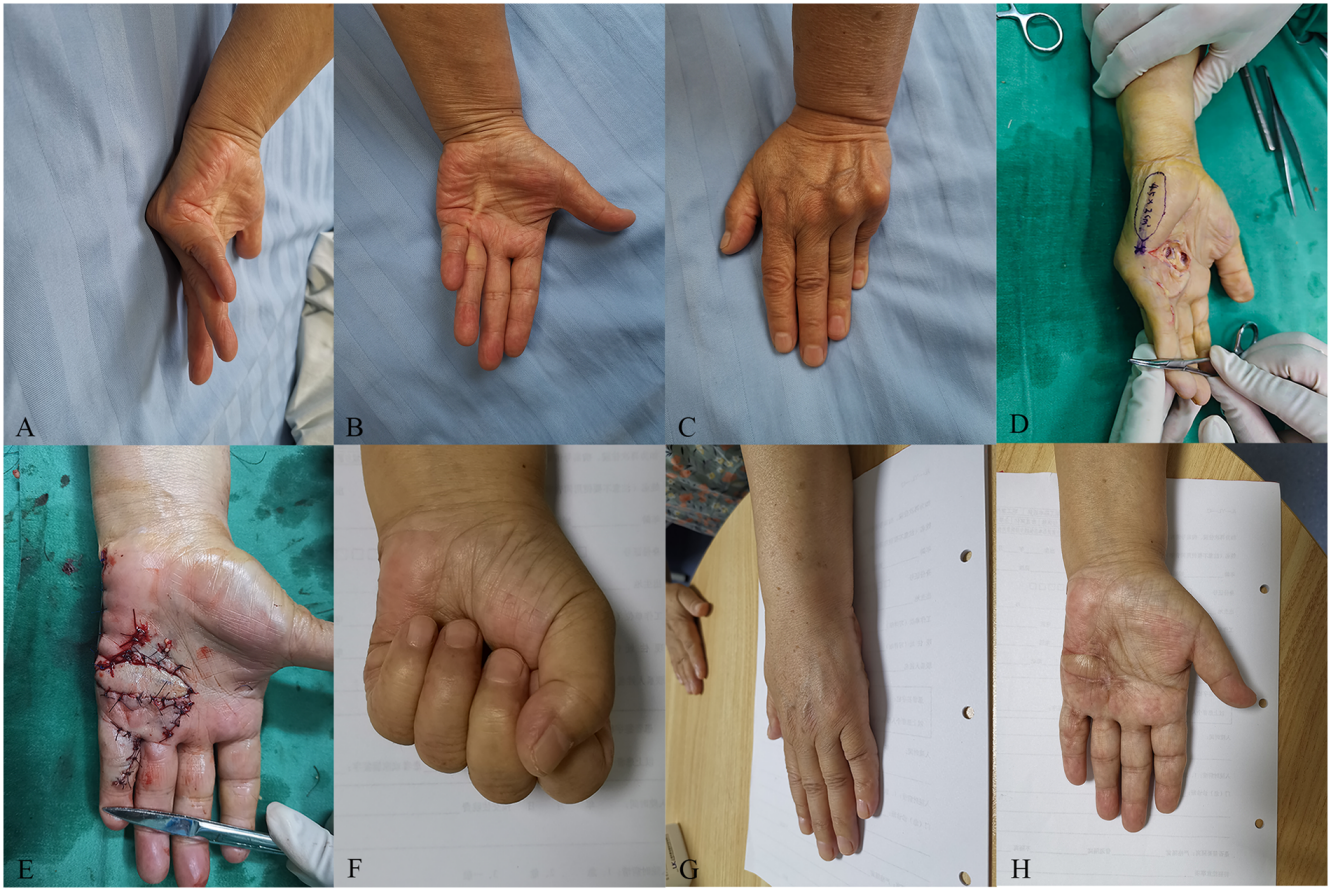
A 73-year-old female patient who suffered from severe Dupuytren’s contracture in her left hand was treated with the perforator flap of the ulnar palm. **(A)** The preoperative MP joint of the ring and little fingers had a flexion angle of 70° and 90°, respectively. **(B)** A cord-like object can be seen on the ulnar palm. **(C)** The patient’s table top test was positive. **(D)** Design of the flap during the operation. **(E)** The wound was covered by the flap and the donor site was closed directly. **(F)** The flap appearance was satisfactory and the active extension of the affected ring and little fingers were normal at the 22-month follow-up. **(G)** The table top test was negative at the 22-month follow-up. **(H)** The active flexion of the affected ring and little fingers was normal at the 22-month follow-up.

A 73-year-old female patient presented to our hospital suffering from severe Dupuytren's contracture in her left palm (Figure 2A). Upon examination, hardened skin was observed on the ulnar side of the palm and the volar side of the proximal section of the fifth digit, along with localized skin depressions. A firm cord-like structure was palpable from the palm to the proximal section of the fifth digit (Figure 2B). The MP joint of the fifth digit had a flexion angle of 90°, the table top test yielded positive results (Figure 2C), the Tubiana stage was classified as III, and the patient opted for surgical intervention.

Prior to surgery, high-frequency Doppler ultrasound was used to locate and mark the perforators of the ulnar palmar digital artery in the fifth digit. The surgery was performed with a tourniquet applied to the brachium (250 mmHg) under a brachial plexus block. Following palmar aponeurotomy and soft tissue release, a palmar wound was created, with the size of 30 mm × 15 mm, exposing the nerves, tendons, and blood vessels. A perforator flap 45 mm in length and 20 mm in width was designed to cover the wound (Figure 2D). The donor site of the flap was directly sutured (Figure 2E). Postoperatively, extension at the MP joint was 0°. The flap survived and the wound healed primarily. At the final follow-up, MP joint extension was maintained at 0°, flexion activity was normal, and the table top test was negative (Figures 2F,G). The patient satisfaction score regarding the flap appearance was 5. The skin color and texture of the flap were similar to the nearby tissue, and there was only a linear scar in the donor area (Figure 2H).

## 4 Results

Of the 25 patients, 20 (80%) were men and 5 (20%) were women with an average age of 60.64 (48–81) years. There were 25 hands involved that were treated with a flap. The size of the soft tissue defects ranged from 1.6 to 3.0 cm (mean, 2.1 cm) in length and 1.5 to 4.4 cm (mean, 3.2 cm) in width. The flaps ranged in dimension from 1.8 cm × 3.0 cm to 2.6 cm × 4.7 cm (mean, 2.4 cm × 3.5 cm). The details of the patients are displayed in Table 1.

**Table 1:** Demographic details of the 25 patients.

Case	Age (years)	Gender	Affected hand	Tubiana stage	Risk factors	Defect size (cm × cm)	Flap size (cm × cm)	Comorbidity
1	73	F	L	III	Diabetes	3.0 × 1.5	4.5 × 2.0	None
2	48	M	L	III	None	2.2 × 3.4	2.4 × 3.7	None
3	65	M	L	III	None	1.8 × 2.0	2.1 × 2.3	None
4	55	M	R	III	None	2.3 × 3.8	2.5 × 4.1	None
5	61	M	R	III	None	2.1 × 3.4	2.4 × 3.7	None
6	60	M	R	III	Smoking	2.0 × 3.3	2.2 × 3.5	Cold sensitivity
7	60	M	L	III	None	1.9 × 2.7	2.2 × 3.0	None
8	56	M	L	III	None	2.2 × 3.3	2.5 × 3.6	None
9	63	F	R and L	III	Diabetes	2.3 × 4.3	2.6 × 4.7	None
10	64	F	L	III	None	2.0 × 3.3	2.3 × 3.6	None
11	68	M	L	III	None	1.8 × 2.0	2.0 × 3.3	None
12	54	M	R and L	III	Diabetes	2.3 × 3.5	2.5 × 3.8	Numbness
13	72	M	R	III	None	1.6 × 2.7	1.8 × 3.0	None
14	55	M	L	III	None	2.2 × 3.7	2.5 × 4.0	None
15	54	M	L	III	None	2.3 × 2.7	2.5 × 3.0	None
16	56	M	R	III	None	2.2 × 3.3	2.5 × 3.6	None
17	55	F	R and L	III	Diabetes	1.9 × 3.4	2.2 × 3.6	None
18	60	M	L	III	None	2.0 × 2.8	2.2 × 3.0	None
19	76	M	R	III	None	2.2 × 3.3	2.5 × 3.6	None
20	60	M	R	III	None	1.7 × 3.4	1.9 × 3.7	None
21	52	M	R	III	Diabetes	2.1 × 4.4	2.3 × 4.7	None
22	48	M	R	III	None	2.0 × 2.7	2.2 × 3.0	None
23	67	F	R	III	Diabetes	2.2 × 3.8	2.4 × 4.0	None
24	53	M	R	III	None	1.8 × 2.8	2.0 × 3.1	None
25	81	M	R	III	None	2.2 × 3.5	2.4 × 3.8	None
Mean						2.1 × 3.2	2.4 × 3.5	

### 4.1 Preoperative data

Preoperative observation showed unilateral and bilateral contractures in 21 (84%) and 4 cases (16%), respectively. Furthermore, 13 patients (52%) presented with contractures in a single digit, while 12 patients (48%) had contractures in multiple digits, most commonly involving the fourth and fifth digits. All patients presented with MP joint flexion contracture, with average values of contracture in the third, fourth, and fifth digits of 86.67°, 87.00°, and 86.32°, respectively.

In addition, 33 digits exhibited PIP joint flexion contracture, with average contracture degrees in the third, fourth, and fifth digits measuring 70.00°, 81.88°, and 74.64°, respectively.

### 4.2 Outcomes of postoperative and final follow-up

The mean follow-up time was 20.48 months (ranging from 15 to 26 months). Immediately postoperatively, the average MP joint flexion contracture degrees in the third, fourth, and fifth digits were 3.33°, 2.50°, and 4.21°, respectively, with complete extension observed at the final follow-up. The average PIP joint flexion contracture degrees in the third, fourth, and fifth digits post-surgery were 1.67°, 7.19°, and 3.93°, respectively. In the final follow-up period, the average PIP joint flexion contracture degrees in the third, fourth, and fifth digits were 1.33°, 3.13°, and 3.21°, respectively. Compared to flexion contracture at the MP joint, flexion contracture at the PIP joint showed less improvement post-surgery. The average values of the MP and PIP flexion contractures preoperatively, immediately postoperatively, and at the final follow-up are shown in Table 2.

**Table 2:** The average flexion contracture of the MP and PIP joints.

Affected digit (°)	Preoperatively		Postoperatively		Final follow-up	
	MP	PIP	MP	PIP	MP	PIP
Third digit	86.67	80.00	3.33	1.67	0.00	1.33
Fourth digit	87.00	81.88	2.50	7.19	0.00	3.13
Fifth digit	86.32	74.64	4.21	3.93	0.00	3.21

MP, metacarpophalangeal joint; PIP, proximal interphalangeal joint.

At the last follow-up, the average MP joint contracture degree in all patients was 0°, matching that of the healthy contralateral hand. Comparison of the PIP joint contracture degrees in the third, fourth, and fifth digits with the healthy hand showed no statistically significant differences (*P* > 0.05) (Table 3).

**Table 3:** The comparison results about the PIP joints of the affected digits and the contralateral digits at final follow-up.

Variable	Third digit	Fourth digit	Fifth digit
*t* ^b^	1.00	1.82	2.10
*P* ^a^	>0.05	>0.05	>0.05

PIP, proximal interphalangeal joint.

^a^ A value of *P* < 0.05 was considered statistically significant.

^b^ Paired-samples *t-*test.

A paired *T*-test was also conducted to compare the recovery of the affected digits at the final follow-up with the degree of contracture before surgery (Table 4). The contracture of the MP joint in the third, fourth, and fifth digits showed a significant difference at the final follow-up compared to the preoperative values (*P* < 0.05). Similarly, the contracture of the PIP joint in the third, fourth, and fifth digits at the final follow-up had a statistically significant difference compared to the preoperative values (*P* < 0.05).

**Table 4:** The comparison results of the affected digits before and after the operation.

Variable	Third digit		Fourth digit		Fifth digit	
	MP	PIP	MP	PIP	MP	PIP
*t* ^b^	26.00	8.37	94.81	13.84	30.21	8.77
*P* ^a^	<0.05	<0.05	<0.05	<0.05	<0.05	<0.05

MP, metacarpophalangeal joint; PIP, proximal interphalangeal joint.

^a^ A value of *P* < 0.05 was considered statistically significant.

^b^ Paired-samples *t-*test.

Based on the results (Table 5), the trend of the patient’s pain severity demonstrated a significant decrease in the final follow-up compared to the preoperative levels (*P* < 0.05). This result suggests the effectiveness of the procedure in treating Dupuytren's contracture. Moreover, the functional status of the affected hands improved following the surgical intervention (*P* < 0.05), which also confirmed the above conclusion.

**Table 5:** Visual analog scale, disabilities of the arm shoulder and hand information.

Variables	Median (*P*25, *P*75)			K–W test	
	Preoperatively	Postoperatively	Final follow-up	*H* ^b^	*P* ^a^
VAS	6 (5, 6)	5 (4, 5)	3 (2, 4)	46.112	<0.05
DASH	65 (50, 70)	45 (30, 50)	25 (20, 30)	58.402	<0.05

VAS, visual analog scale; DASH, disability of the arm, shoulder, and hand.

^a^ A value of *P* < 0.05 was considered statistically significant.

^b^ Kruskal–Wallis test.

Upon final assessment, the hands exhibited an esthetically pleasing appearance with only linear scars visible in the donor flap and flap areas, matching the color and texture of the surrounding skin. The table top tests of the patients were negative. The average two-point discrimination value of the flap was 5.52 ± 0.97 mm. The results of the TAM assessment were classified as excellent in 19 cases and good in 6 cases, yielding an average TAM of 229° ± 16.75°. The outcomes of the MHQ indicated that 19 cases reported being very satisfied with the appearance, 5 cases were satisfied, and 1 case was deemed normal. The average satisfaction score for the skin flap appearance was 4.72.

## 5 Discussion

There is currently no cure for Dupuytren's contracture, and the treatment focuses on improving hand function by reducing joint contracture. In the early stages of the disease, various non-surgical treatments can be employed, such as drug therapy with vitamin E or steroids, and non-invasive options such as physical therapy and radiation (9, 10). For late-stage disease, a broader array of treatment options is available, including percutaneous needle fasciotomy (PNF), collagenase *Clostridium histolyticum* (CCH) injection, or surgical excision to remove the diseased tissue through limited fasciectomy (LF) or cutaneous fasciectomy.

Research indicates that non-invasive treatments for Dupuytren's contracture have limited effectiveness and are often associated with a high recurrence rate (11). In contrast, invasive procedures are linked to a lower risk of recurrence, although they necessitate a longer postoperative recovery period.

Multiple studies have demonstrated that PNF improves flexion deformities and allows for quicker recovery post-surgery compared to fasciectomy; however, it has a higher 5-year recurrence rate of approximately 30%, compared to 6% for limited fasciectomy (12). The PNF technique is considered safe, although potential complications include flexor tendon rupture and nerve damage. As a minimally invasive therapy, CCH injection is easy to perform and facilitates faster postoperative recovery with minimal disruption to the patient’s daily activities. Nonetheless, complications such as hematoma, nerve injury, and impaired wound healing have been observed, with an incidence rate of 23% (13). A randomized controlled trial comparing PNF and CCH injections found that PNF is comparable to CCH in correcting flexion and contraction deformities, although with a potentially higher risk of recurrence (14).

The current standard treatment is LF (15). It is associated with a high complication rate (approximately 20.9%) over the subsequent 5 years due to increased wound skin tension and larger scars (16, 17). To address this issue, flap coverage of soft tissue defects due to LF is being considered as it may reduce surgical risks and recurrence rates while maintaining the benefits of the resection. Commonly utilized skin extension techniques for the surgical treatment of Dupuytren's contracture include YV-plasty and Z-plasty. However, these methods may prove challenging for achieving adequate skin extension in cases where the preoperative contracture is severe, the skin defect is extensive, the defect is difficult to cover, or when the PIP contracture exceeds 30° (18). Following YV- or Z-plasty, the tension in the skin after the release of the contracted joint may be excessively high. Patients with severe Dupuytren's contracture often present challenges in achieving complete extension through conventional surgical techniques, with insufficient skin extension being a significant contributing factor (19). This lack of skin extension not only impedes early active recovery in the direction of extension but also diminishes the overall effectiveness of postoperative recovery. In light of this issue, surgeons frequently opt to utilize flaps to cover the soft tissue defect following a fasciectomy, thereby addressing the problem of inadequate skin extension that arises from direct suturing. The use of the first dorsal metacarpal artery island flap for repairing soft tissue defects from Dupuytren's contracture may not be ideal for men with hairy skin on the back of their hands (20), as the skin on the back of the hand differs esthetically and sensuously from the palmar skin, with variations in touch and tactile corpuscle density (21, 22). When utilizing the first dorsal metacarpal artery island flap to address soft tissue defects caused by LF, the resulting appearance and texture of the treated area may differ from that of unaffected sites. The free forearm flap is effective for repairing hand defects; however, it imposes high technical demands on the operator and may result in significant defects at the donor site (23). In addition, the excess tissue of the excised skin flap may necessitate a secondary procedure for reduction. In some instances, the thenar flap has been utilized to transpose and suture palm defects caused by the operation (24). It is important to recognize that Dupuytren's contracture predominantly affects the ulnar side, with higher contracture rates observed in the ring finger and little finger compared to the thumb and index finger (25). Following the principle of proximity in skin flap repair, a perforator flap from the ulnar palm presents a viable option for addressing soft tissue defects on the distal palm and the palmar aspect of the little finger (6).

The design of the flap is simple, the flap donor area is easy to close, the flap blood supply is good, and the flap is rich in adipose tissue and does not easily atrophy after the operation (26). Researchers such as Bakhach et al. have leveraged the dorsal blood supply of the ulnar artery to opt for an ulnar parametacarpal flap (27, 28). Other researchers such as Hirase et al. have successfully employed the ulnar palmar digital artery as a perforator (29), yielding satisfactory outcomes in treating soft defects on the palmar side of the little finger. Notably, this flap can also serve as a rotational flap to cover the palmar ulnar MP joints (29). In addition, the position of the efferent deep fascia of the perforator branch emanating from the ulnar palmar artery is relatively constant. It penetrates between the superficial hypothenar muscles and the fifth metacarpal bone to the subcutaneous tissue and anastomoses with the small blood vessels emanating from the descending dorsal branch of the ulnar artery to form the vascular chain nutrient flap, which increases the possibility of the survival of the flap and enlarges the size of the flap. In cases where the longitudinal scar contracture in Dupuytren's contracture involves the skin of the fingers, flexion contracture is likely to occur. The flap is harvested from the skin on the lateral aspect of the ulnar palm, minimizing contact with the contracture tissue of the Dupuytren's contracture. This creates a transverse barrier across the palm, effectively reducing the recurrence rate (30). Given that the donor area of the flap was situated on the volar ulnar side, it is essential to pay careful attention to the palmar-dorsal junction, which is located away from the hypothenar region, during the flap removal process. This precaution is necessary to prevent complications such as grip discomfort or sensory loss following the operation (31). The flap used in this procedure provides skin that closely resembles the palm in texture, appearance, and feeling (32). It allows for a relatively long flap to be harvested compared to other flaps in the hand (33), but may not be suitable for large wounds (34). This flap is effective for repairing medium-sized defects, particularly when the flap width does not exceed 25 mm, allowing for direct closure of the donor site with minimal scarring (34). A previous study suggests that the choice of a non-fingertip defect reconstruction flap depends on the defect size, with small defects being less than 4 cm^2^, medium defects ranging between 4 and 8 cm^2^, and large defects usually exceeding 8 cm^2^ (35). The ulnar palmar flap can be used to cover the flexor tendons and neurovascular bundles, thus preventing flexor tendon adhesions and neurological impairment. However, caution must be taken to prevent venous congestion when using this flap (36). Post-surgery, flap monitoring, and adherence to rehabilitation nursing are recommended. Using a heat lamp may help to promote blood circulation in the flap and improve its survival rate. It is advised to engage in appropriate rehabilitation exercises with the aid of professional physical therapists.

The advantages of the flap include a reliable blood supply, matching skin texture, a thin flap, absence of swelling in the recipient area, esthetic appeal, and no adverse effects on hand grip function. In addition, the flap causes minimal collateral damage due to direct suturing in the donor area, resulting in only a slight linear scar on the wound surface. It is also easy to obtain and provides effective coverage for palmar defects without significant functional impairment or complications in the upper limb. However, it is not suitable for wounds on the middle finger or large-sized defects.

The limitations of this study include the following aspects. First, it employs a retrospective design with a relatively small sample size, which presents certain constraints. Second, the absence of a control group limits the ability to compare the advantages of this treatment with other methods through specific data analysis. Third, the follow-up period is short; further long-term follow-up is necessary to assess the effectiveness of this flap in treating skin defects caused by Dupuytren's contracture and to evaluate the incidence of long-term complications. Fourth, patient-reported evaluations may influence the actual outcomes. Finally, the patient population is limited in terms of ethnicity.

## 6 Conclusion

The perforator flap of the ulnar palm is particularly effective for skin and soft tissue defects on the ulnar side of the palm or the palmar side of the fifth digit, making it suitable for treating severe Dupuytren's contracture. Based on the discussion of the advantages of this flap and the limitations of this study, it is necessary to conduct a comparative study and long-term follow-up of this flap and other methods for the treatment of skin defects caused by surgical treatment of Dupuytren's contracture.

## Data Availability

The original contributions presented in the study are included in the article/Supplementary Material, further inquiries can be directed to the corresponding author.
